# Using simulation to accelerate autonomous experimentation: A case study using mechanics

**DOI:** 10.1016/j.isci.2021.102262

**Published:** 2021-03-02

**Authors:** Aldair E. Gongora, Kelsey L. Snapp, Emily Whiting, Patrick Riley, Kristofer G. Reyes, Elise F. Morgan, Keith A. Brown

**Affiliations:** 1 Department of Mechanical Engineering, Boston University, Boston, MA 02215, USA; 2 Department of Computer Science, Boston University, Boston, MA 02215, USA; 3 Google Research, Mountain View, CA 94043, USA; 4 Department of Materials Design and Innovation, University at Buffalo, Buffalo, NY 14260, USA; 5 Department of Biomedical Engineering, Boston University, Boston, MA 02215, USA; 6 Division of Materials Science & Engineering, Boston University, Boston, MA 02215, USA; 7 Physics Department, Boston University, Boston, MA 02215, USA

**Keywords:** Mechanical Property, Computational Method in Materials Science, Simulation in Materials Science

## Abstract

Autonomous experimentation (AE) accelerates research by combining automation and machine learning to perform experiments intelligently and rapidly in a sequential fashion. While AE systems are most needed to study properties that cannot be predicted analytically or computationally, even imperfect predictions can in principle be useful. Here, we investigate whether imperfect data from simulation can accelerate AE using a case study on the mechanics of additively manufactured structures. Initially, we study resilience, a property that is well-predicted by finite element analysis (FEA), and find that FEA can be used to build a Bayesian prior and experimental data can be integrated using discrepancy modeling to reduce the number of needed experiments ten-fold. Next, we study toughness, a property not well-predicted by FEA and find that FEA can still improve learning by transforming experimental data and guiding experiment selection. These results highlight multiple ways that simulation can improve AE through transfer learning.

## Highlights

• Simulation and autonomous experimentation were combined to accelerate research
• Resilience, which was accurately simulated, was learned in 10 x fewer experiments
• Simulating related properties, i.e. yield force, accelerated learning toughness
• Simulation was introduced to an experimental learning loop using transfer learning

## Graphical abstract

**Figure:**
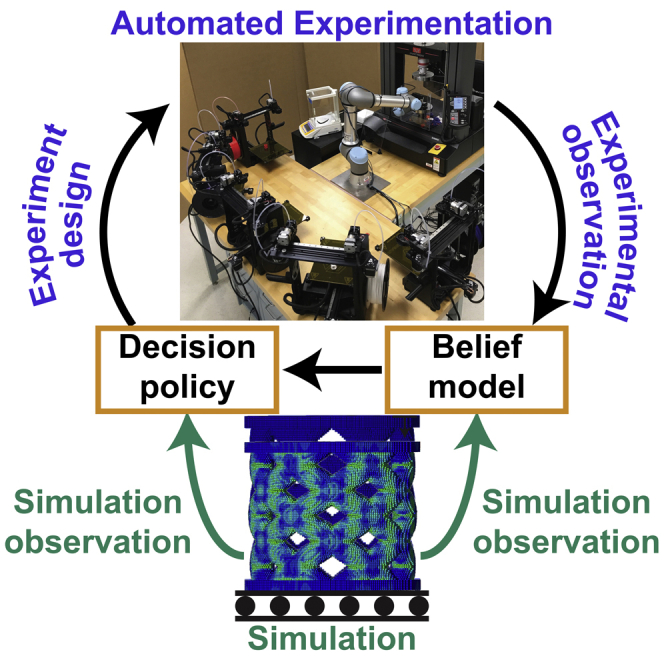

## Introduction

Designing materials and structures with optimized properties is a paramount goal of materials science and engineering (Wegst et al., 2015; Yeo et al., 2018). For instance, successes in the study of architected materials have shown that modifying the geometry of lattice-like structures is a powerful method for tuning mechanical properties. Key to the exploration of such intricate structures are advances in high-performance computing and simulation methods, namely finite element analysis (FEA), that have enabled the computation of many facets of mechanical performance (Bar-Sinai et al., 2020; Gao et al., 2003; Kochmann and Bertoldi, 2017). By combining FEA with optimization algorithms, approaches such as topology optimization (Barthelat and Mirkhalaf, 2013; Boddeti et al., 2018; Chen et al., 2018; Jin et al., 2020; Sigmund and Maute, 2013) have led to discovery of intriguing hierarchical structures and composites.

While simulation is powerful, it cannot predict all aspects of mechanical performance, necessitating physical experiments. Mechanics is one of many fields in which experiments can present a bottleneck to progress, a challenge that has motivated the development of autonomous experimentation (AE) systems in numerous fields such as biology (Bryant et al., 2004; King et al., 2009), materials science (MacLeod et al., 2020; Nikolaev et al., 2016; Noack et al., 2019), chemistry (Bédard et al., 2018; Burger et al., 2020; Epps et al., 2020; Porwol et al., 2020), and mechanics (Gongora et al., 2020) to efficiently explore vast and multi-dimensional parameter spaces without human intervention. Ultimately, AE accelerates research by utilizing automation to perform experiments rapidly and using machine learning to select experiments that yield best progress toward the chosen goal. As such, many AE-related advances have involved improved automation (Coley et al., 2019; Li et al., 2018; Nikolaev et al., 2014; Ren et al., 2018; Sun et al., 2019) or algorithms (Wang et al., 2015). Improving collection of experimental data has been critical in this effort, because the premise that simulation is imperfect has led the community to largely proceed in an experimentally data-driven fashion. While this premise is not incorrect, simulation can still in principle provide value for experimental campaigns. A remaining open question, whose answer likely depends upon the relationship between simulation and experiment, is how best to incorporate simulation into AE.

**Figure 1:**
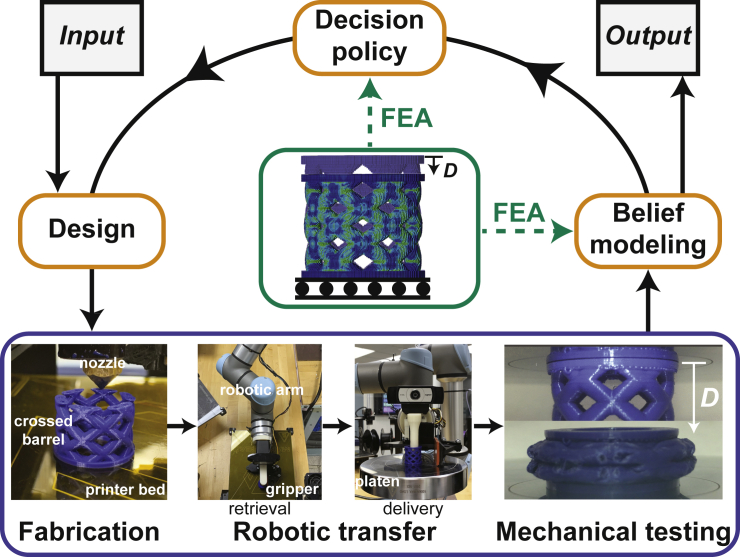
Incorporating finite element analysis (FEA) into a Bayesian experimental autonomous researcher (BEAR) to study the mechanical behavior of additively manufactured components

Here, we test the hypothesis that incorporating knowledge from simulation with AE can accelerate the pace of research in the context of mechanics (Figure 1). To explore this concept, we use a robotic system both to 3D-print components and to test them in uniaxial compression (Gongora et al., 2020). When combined with a Bayesian optimization (BO) algorithm to iteratively select experiments that will maximize a performance metric such as component toughness, this system is termed a “Bayesian experimental autonomous researcher” (BEAR). To understand how FEA can improve the operation of the BEAR, we first compare FEA predictions to experimental measurements and determine that while resilience is well predicted by FEA, toughness is not. We then explore the use of the BEAR to optimize resilience by using discrepancy modeling and FEA in the belief model. We evaluate this approach by conducting experimental campaigns and find that, as compared to BO with an uninformative prior, using FEA in this fashion can reduce by a factor of ∼10 the number of experiments necessary to find high-performing structures. Finally, we study BEAR campaigns to optimize toughness using a custom method in which the belief model is built on FEA-transformed data and the decision policy is FEA-informed. We find that these FEA-informed experimental learning campaigns resulted in ∼15% higher performing structures compared to campaigns using a traditional BO approach. For the evolving field of AE and data-driven research more broadly, this work shows the potential for capitalizing on additional information sources such as simulation to accelerate the pace of research and enable the exploration of more complex parameter spaces.

## Results and discussion

### Comparison of simulated and measured mechanical properties of parametric structures

**Figure 2:**
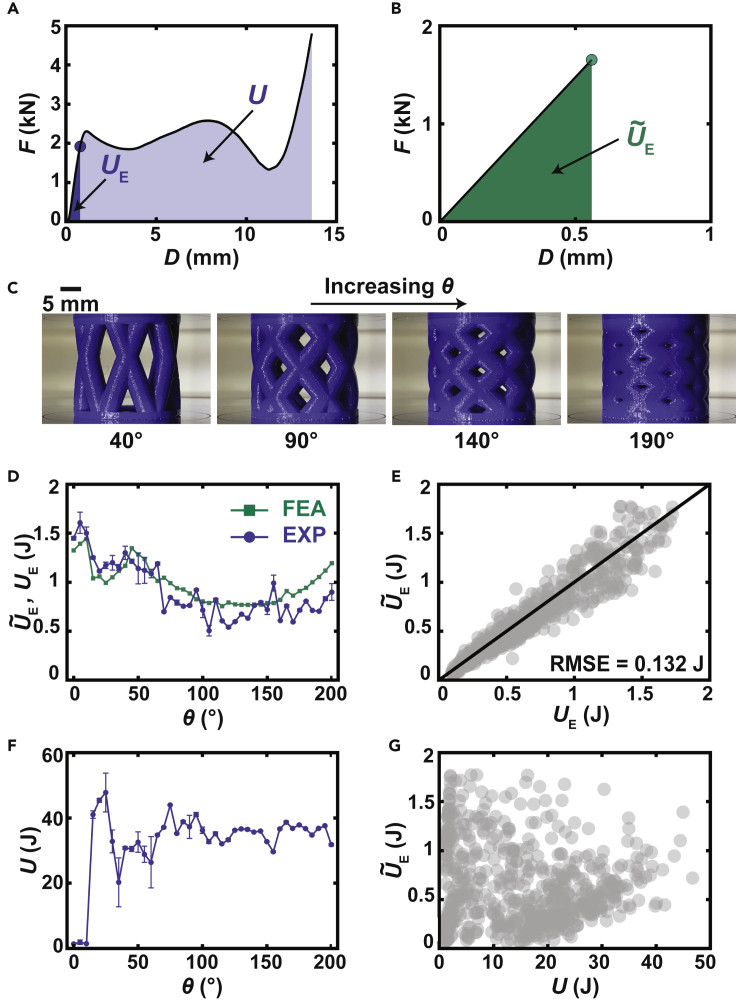
Exploration of resilience and toughness of parametric structures (A) Force *F* vs. displacement *D* for a crossed barrel structure measured using quasi-static compression. Resilience $U_{E}$ and toughness U are computed as areas under the *F*-*D* curve as shown. (B) *F* vs. *D*. computed using finite element analysis (FEA) along with simulated resilience ${\tilde{U}}_{E}$ found as the area under the *F*-*D* curve. (C) Photographs of a series of crossed barrel structures that vary in their twist angle θ. (D) $U_{E}$ and ${\tilde{U}}_{E}$ vs. θ for a series of crossed barrels including those depicted in (C). While this plot highlights the dependence of resilience on θ as a single parameter, it is worth emphasizing that we are exploring a four-dimensional parameter space. Points denote mean with error bars denoting standard deviation. (E) ${\tilde{U}}_{E}$ vs. $U_{E}$ for 600 crossed barrels selected in a grid across the entire four-dimensional parameter space. Agreement between the quantities is evident based upon the root mean square error (RMSE). (F) U vs. θ for a series of crossed barrels including those depicted in (C). Points denote mean with error bars denoting standard deviation. (G) ${\tilde{U}}_{E}$ vs. U for 600 crossed barrels selected in a grid across the entire four-dimensional parameter space. These properties are found to be uncorrelated.

In order to determine the degree to which FEA could predict the mechanical behavior of additively manufactured structures, we evaluated a “crossed barrel” family of components that leverage our previously reported dataset (Gongora et al., 2020). In the previously reported data set, the “crossed barrel” family of components is parametrized by n hollow columns with outer radius r and thickness t that are twisted with an angle θ. Thus, the four-dimensional parameter space considered in this work is defined by x=(n,θ,r,t). In a typical experiment, a component was printed out of polylactic acid (PLA) filament and tested in uniaxial, quasi-static compression (Figure 2A). This experiment allowed direct computation of two important metrics of energy absorbed by a component during compression, namely toughness U and resilience $U_{\text{E}}$. Resilience is defined as the energy stored during the elastic portion of the compressive curve while toughness is defined as the energy absorbed during the entirety of the compression (elastic and failure). Optimizing the former is important for realizing structural components that accommodate a variety of working conditions without damage while optimizing the latter is critical for realizing structures that are safe during catastrophic events. From the design of the “crossed barrel” structure used for 3D printing, a mesh generated from hexahedral elements (Figures S1 and S2) can be used in FEA to predict resilience ${\tilde{U}}_{\text{E}}$ by simulating a uniaxial quasi-static compression test (Figure 2B).

To assess the prediction capabilities of FEA, experimental measurements and FEA predictions were obtained for a series of components that varied based upon a single parameter, namely the twist angle θ (Figure 2C). The root mean squared error (RMSE) between $U_{\text{E}}$ and ${\tilde{U}}_{\text{E}}$ for the series of components was 0.1801 J (Figure 2D). Perhaps more importantly, the FEA predictions reasonably captured the general trends present in the experimental measurements. Not only did $U_{\text{E}}$ and ${\tilde{U}}_{\text{E}}$ exhibit similar dependencies on θ but they also showed excellent agreement with each other for 600 distinct designs spread across the four-dimensional parameter space, exhibiting RMSE = 0.132 J and a mean squared percentage error of 3.12% (Figure 2E).

Toughness could not be computed due to the need for painstaking management of self-contacts and development of advanced material models (Li et al., 2017; Qiao et al., 2008; Zhu et al., 2010) which limit the throughput of computation. While one could expect that FEA predictions of resilience could provide some value as resilience contributes to toughness by definition (*i*.*e*. Figure 2A), examining U for this class of structures shows that U and ${\tilde{U}}_{\text{E}}$ are not similar in magnitude (Figure 2F). This difference is rooted in mechanics with the energy absorbed during plastic deformation not being directly correlated with that stored during elastic deformation. However, there are some structures for which $U\approx U_{E}$, namely those that exceed the force threshold and therefore never enter the plastic regime during the working range (*i*.*e*. those for which θ≤15°). Nevertheless, the disagreement between these quantities manifests across the entire parameter space with ${\tilde{U}}_{\text{E}}$ not being correlated with U($R^{2}=0.0133$) (Figure 2G). It should be noted that the lack of correlation between $U_{E}$ andU is likely a general property of lattice-based structures as these feature a vast diversity of behaviors that only begin once the structure enters the plastic regime.

### Optimizing resilience with an FEA-informed BEAR

In this work, we employed BO due to its popularity and previously reported success as an active learning strategy for optimization. Additionally, active learning approaches, such as BO, have been previously reported to outperform one factor at a time (OFAT) and design of experiment (DoEs) approaches (Braham et al., 2020). In particular, OFAT can be slow and inefficient in high-dimensional parameter spaces since a single variable is varied at a time. Further, OFAT is highly sensitive to the initial selection of variables and does not rapidly capture potential correlations between input variables. While DoE approaches address some of the aforementioned shortcomings of OFAT, they depend on an initial round of experiments being conducted before analysis can proceed (Bowden et al., 2019). Additionally, DoE approaches are unable to efficiently capture highly non-linear parameter spaces and when applied iteratively tend to be generally exploitative (Cao et al., 2018). Active learning approaches, such as BO, improve upon OFAT and DoE by using all available experiments to build belief models that can capture complex and highly non-linear parameter spaces. Moreover, active learning approaches enable the iterative selection of subsequent experiments using decision policies that can determine and exploit correlations between input variables, and they can be customized to favor exploration, exploitation, or balance these two goals (Lookman et al., 2019; Rohr et al., 2020).

Given that FEA can reasonably predict resilience, learning or optimizing experimental resilience can be considered a classic transfer learning process (Pan and Yang, 2010). Specifically, both $U_{\text{E}}\left(x\right)$ and ${\tilde{U}}_{\text{E}}\left(x\right)$ are defined over the same parameter space – namely the four-dimensional space corresponding to the parametric family of structures given by x=(n,θ,r,t). To define ${\tilde{U}}_{\text{E}}\left(x\right)$ over the parameter space, we built a surrogate model from FEA predictions selected on a grid (Figures S3 and S4A). Since ${\tilde{U}}_{\text{E}}\left(x\right)$ represents an approximation for $U_{\text{E}}\left(x\right)$, the optimization problem can be addressed using relational knowledge transfer, an approach in inductive transfer learning. Specifically, we define a discrepancy model $\delta_{E}\left(x\right)=U_{\text{E}}\left(x\right)-{\tilde{U}}_{\text{E}}\left(x\right)$ to represent the difference between FEA and experiment. This approach differs from BO with an uninformative prior in that, rather than selecting experiments based upon a belief model of $U_{E}$ that is a Gaussian process regression (GPR) against experimental measurements of $U_{E}$, the discrepancy model approach uses a GPR trained on $\delta_{E}=U_{\text{E}}-{\tilde{U}}_{\text{E}}$. In other words, the FEA-informed approach makes ${\tilde{U}}_{\text{E}}$ the Bayesian prior for the belief model.

**Figure 3:**
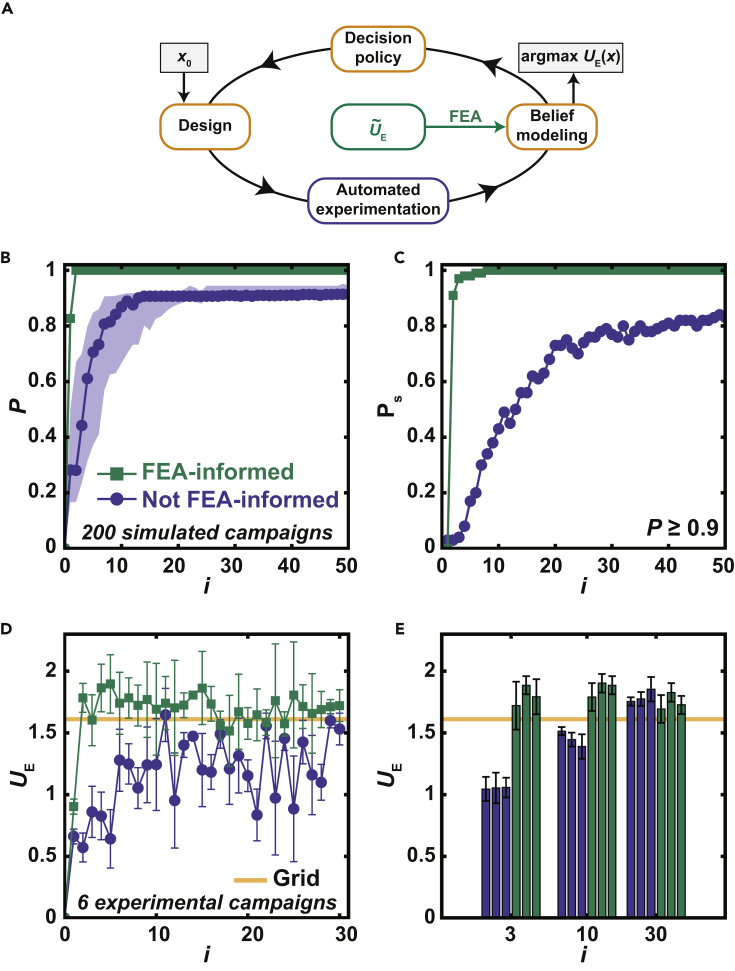
Combining AE and simulation to optimize resilience (A) Scheme showing how FEA was incorporated into the BEAR to find the design *x* with the maximum *U*_E_. The process begins with a random design *x*_0_. (B) Simulated performance *P* at experiment number *i* for a simulated learning campaign. (C) Probability of achieving P≥ 0.90 at a given *i*. (D) Sequence of *U*_E_ measured during six experimental learning campaigns. Points denote mean with error bars denoting standard deviation. The horizontal line indicates the maximum value measuring on a grid of 600 points. (E) *U*_E_ measured at the optimum predicted for each of the six campaigns after *i* experiments. Each value represents the average of five identically prepared samples with the error bars denoting their standard deviation.

In order to evaluate the incorporation of FEA into a BEAR through discrepancy modeling (Figure 3A), we performed a series of simulated learning campaigns based upon an expected improvement (*EI*) decision policy using either the uninformative prior or the FEA-informed approach. The *EI* decision policy was selected due to its widespread application in the BO community as an improvement-based decision policy that seeks to select subsequent experiments based on the likelihood of exceeding previous observations. Since the simulated learning campaigns are *in silico*, we may evaluate the performance Pof these approaches using P=1 to indicate that the campaign found the optimum design, or the *x* that corresponded to the largest $U_{E}$ in the data set. Campaigns using the FEA-informed approach achieved a median performance Md(P)=1 in only two experiments, whereas campaigns using an uninformative prior only achieved Md(P)≥0.90 after 13 experiments (Figure 3B). To further assess these approaches, we computed the probability ${\text{P}}_{s}$ of achieving P≥0.90 where ${\text{P}}_{s}\;=\;1$ would indicate that all simulated campaigns had achieved P≥0.90. Campaigns using an FEA-informed approach achieved ${\text{P}}_{s}\;\geq$ 0.99 after six experiments, while those using an uninformative prior only achieved ${\text{P}}_{s}=0.80$ after 32 experiments (Figure 3C). These results clearly predict that a good prior from simulation will substantially accelerate an experimental campaign.

To estimate how sensitive the improvement was to the accuracy of the FEA predictions, we first repeated the FEA computations while varying E and $\sigma_{y}$ to determine the degree to which these input material properties influenced ${\tilde{U}}_{E}$. Interestingly, ${\tilde{U}}_{E}$ is more than twice as sensitive to errors in $\sigma_{y}$ than errors in E, with overestimations of $\sigma_{y}$ increasing ${\tilde{U}}_{E}$ and overestimations of E decreasing ${\tilde{U}}_{E}$ (Figures S5A and S5B). To determine the degree to which such errors would affect a learning campaign, we performed a series of simulated learning campaigns in which the FEA results were multiplied by a factor $\varphi_{FE}$ = 0.50, 0.90, 0.95, 1.05, 1.1, and 1.5. Variations in the FEA results that were ∼10% (which correspond to a ∼7% error in $\sigma_{y}$ or a ∼28% error in E), had a minimal effect on convergence (Figures S5C and S5D). Larger errors, however, had a more substantial impact on convergence with the interesting result that overestimations in FEA results were more damaging to learning than underestimations (Figures S5E and S5F). We hypothesize that this asymmetry is due to the goal of the algorithm being to maximize resilience.

Guided by these simulated learning campaigns, we performed six independent experimental learning campaigns to further assess the incorporation of FEA into a BEAR through discrepancy modeling (Table 1). Three campaigns used an FEA-informed approach, and three used an uninformative prior. Each campaign was given an experimental budget of 30 experiments. By comparing the average $U_{E}$ for each approach as a function of experiment number, the FEA-informed approach on average outperformed the uninformative-prior approach (Figure 3D). Additionally, the average resilience using the FEA-informed approach was mostly larger, after one experiment, than the mean of the predicted maximum resilience of 1.61±0.17 J from a GPR surrogate model trained on resilience measurements at 600 distinct design locations specified in a grid search (“Grid”).

**Table 1:** Mean and standard deviation of resilience after 3 experiments for both the uninformative-prior approach (not FEA-informed) and the FEA-informed approach

Approach	Mean $U_{E}$ (J)	SD (J)
Not FEA-informed	1.05	0.10
	1.05	0.12
	1.06	0.08
FEA-informed	1.72	0.19
	1.89	0.07
	1.79	0.14

It is key to note that BO campaigns with EI attempt to balance both exploration and exploitation, and thus not all subsequent experiments will yield an increase in the experimental response. While the sequence of experimental responses can provide some insight into performance, it is imperative to assess performance by evaluating the experimental response of the predicted optimum design of the belief model after i experiments. To do this, we carried out five repeated experiments at the predicted optimum designs of each of the experimental campaigns after three, 10, and 30 experiments (Figure 3E). After three experiments, the FEA-informed approach outperformed the uninformative-prior approach by ∼71% and the Grid result by ∼12%. After 10 experiments, the performance of the uninformative prior approach increased but was still ∼28% less than the performance of the FEA-informed approach. After 30 experiments, the performance of the uninformative prior approach and the FEA-informed approach were not statistically different (p=0.39), while both approaches outperformed Grid by ∼10%. Additionally, the performance of the FEA-informed approach after 30 experiments was not statistically different than the performance of the FEA-informed approach after 10 experiments based on a multiple comparison analysis comparing each campaign (p > 0.05). Ultimately, the FEA-informed approach reduced the number of experiments necessary to find a high-performing design by 10-fold relative to the uninformative prior approach and by 600-fold relative to Grid. Notably, campaigns based upon the uninformative prior approach found better designs in 30 experiments than resulted from the 1800 experiments used as part of the Grid. The observed 60-fold reduction in number of experiments recapitulates our previously reported acceleration of BO relative to grid searching when optimizing toughness (Gongora et al., 2020).

### Optimizing toughness with an FEA-informed BEAR

**Figure 4:**
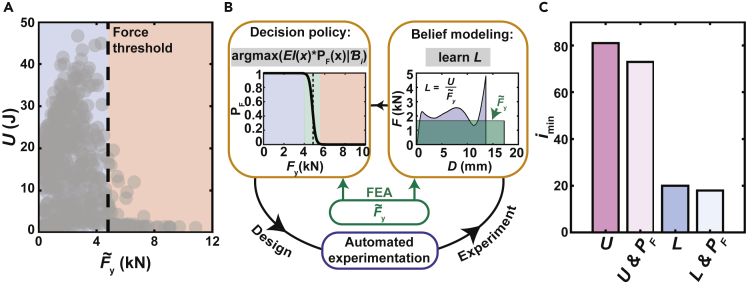
Incorporation of a belief model built on FEA-transformed data and an FEA-informed decision policy into a BEAR (A) *U* vs. FEA-predicted yield force ${\tilde{F}}_{y}\;$for 600 distinct designs selected on a grid. The dashed line corresponds to the experimental force threshold. (B) Schematic showing how FEA was incorporated into the BEAR. The decision policy was modified using a logistic function $P_{F}$. The experimental data was transformed into an effective length L using ${\tilde{F}}_{y}$. (C) Estimate of the minimum number of experiments *i*_min_ needed to explore parameter space using strategies based on U, U and $P_{F}$, L, and L and $P_{F}$.

While ${\tilde{U}}_{E}$ was clearly useful in optimizing $U_{E}$, its lack of correlation with U makes its utility in a toughness optimization framework substantially less clear. Indeed, we performed simulated campaigns exploring the simplest extension of the resilience studies by building a Bayesian prior with ${\tilde{U}}_{E}$ and using discrepancy modeling to find the inelastic component of toughness (*i*.*e*. *U* - *U*_E_). Unfortunately, simulation suggested that this type of knowledge transfer would not provide any acceleration, indicating that more creative approaches to using FEA are needed. One such approach is motivated by how tough structures are used in practice. Specifically, tough components are often accompanied by a force threshold to allow them to absorb energy before transmitting dangerous reactive forces to other elements in a system. With this force criterion in mind, the FEA-predicted yield force ${\tilde{F}}_{y}$ (Figure S4B) becomes a very useful factor as it can differentiate between structures whose design allows them to plastically deform during failure and those that are too strong for the imposed force threshold. For our data set, while a low ${\tilde{F}}_{y}$ did not guarantee high performance, structures with high ${\tilde{F}}_{y}$ were all low-performing (Figure 4A). To leverage this realization, we constructed a logistic function ${\text{P}}_{\text{F}}\left(x\right)$ trained using ${\tilde{F}}_{y}$ that biases the system away from regions of parameter space that are too strong, thus shrinking parameter space by ∼9% (Figure 4B). Specifically, ${\text{P}}_{\text{F}}$ was built using ${\tilde{F}}_{y}$ computed for 1188 designs selected on a grid, where ${\text{P}}_{F}=1$ indicated that ${\tilde{F}}_{y}$ would not exceed the force threshold and ${\text{P}}_{\text{F}}=0$ indicated that it would. The transition between 0 and 1 was given a width of 15%, a number chosen to match the median coefficient of variation of U. Subsequently, ${\text{P}}_{\text{F}}$ was incorporated into the decision policy by selecting the next experiment by finding $\text{argmax}\left(EI\left(x\right)\cdot {\text{P}}_{\text{F}}\left(x\right)\right)$ (Gelbart et al., 2014). This approach effectively filters the parameter space by removing designs that are predicted to be low-performing.

In parallel, we explored a way to reduce the *effective* size of parameter space by using simulation data to transform experimental data into a format that featured greater correlations in parameter space. Specifically, we defined an effective length $L\left(x\right)\;=\;U\left(x\right)/{\tilde{F}}_{y}\left(x\right)$ that represents how much compression would be required at constant ${\tilde{F}}_{y}$ to produce the same toughness as the experimentally determined value U. A belief model of L was built using a GPR, and then this model was combined with ${\tilde{F}}_{y}$ to form the input to the decision policy (Figure 4B).

In order to estimate whether these approaches—one that uses an FEA-informed decision policy and one that uses a belief model build on FEA-transformed experimental data—would reduce the number of experiments needed to explore the parameter space, we hypothesized that examining the correlation lengths of GPRs trained on these datasets would provide insight. In particular, longer correlation lengths would indicate that each data point is providing information relevant to larger regions in parameter space. To explore this systematically, we divided the total volume of parameter space by the product of these correlation lengths to approximate how many experiments would be needed to explore space (Figure 4C). As expected, shrinking the parameter space by ∼9% based on ${\text{P}}_{F}$ commensurately reduced the number of needed experiments. Strikingly, learning L rather than U was predicted to produce a four-fold reduction in the number of needed experiments. We hypothesize that this reduction could be explained by the physical processes at play. Specifically, since toughness is both a product of the strength of the component and its ductility, factoring out a representation of its strength could remove one source of variability, allowing experiment to focus on learning one quantity – ductility – more directly.

**Figure 5:**
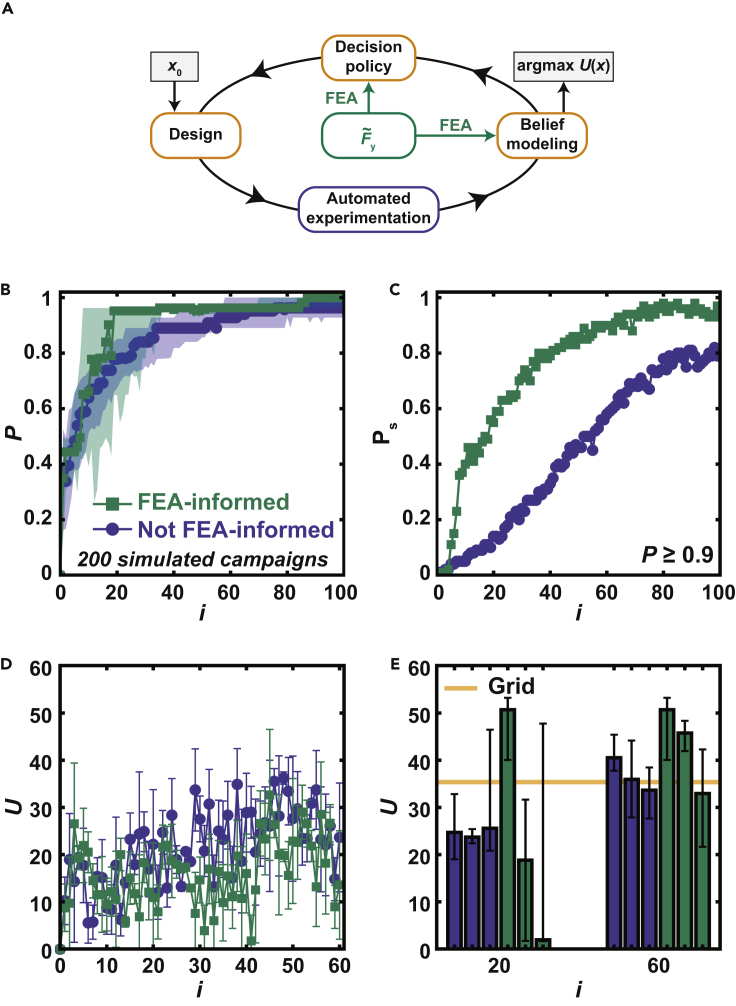
Optimizing toughness of parametric structures (A) Strategy for incorporating FEA into the BEAR through a belief model built on FEA-transformed data and an FEA-informed decision policy. (B) Simulated performance *P* at experiment number *i* for a simulated learning campaign to estimate toughness. (C) Probability $P_{s}$ of achieving P≥ 0.90 at a given *i*. (D) Sequence of *U* measured during six experimental learning campaigns. Points denote mean with error bars denoting standard deviation. (E) *U* measured at the optimum predicted for each of the six campaigns after *i* experiments. Each value represents the median of five identically prepared samples with the error bars denoting their total range.

We next evaluated the incorporation of these two approaches, termed together as an “FEA-informed approach,” into a BEAR (Figure 5A). We performed a series of simulated learning campaigns using the uninformative-prior approach and the FEA-informed approach to optimize U defined over the four-dimensional parameter space x=(n,θ,r,t). Campaigns using the FEA-informed approach achieved Md(P)≥0.95 after 19 experiments, outperforming campaigns using an uninformative prior which achieved a Md(P)≥0.95 after 66 experiments (Figure 5B). Interestingly, the interquartile range of the FEA-informed campaigns was notably reduced after 36 experiments. Additionally, the FEA-informed approach achieved Md(P)=1 after 87 experiments while the uninformed prior approach plateaued at Md(P)=0.96 after 80 experiments. The campaigns based upon the FEA-informed approach reached $P_{s}\;\geq 0.85$ after 50 experiments and an average $P_{s}\;>0.90$ after 60 experiments, outperforming campaigns using an uninformative prior which only achieved $P_{s}\;\geq 0.58$ after 60 experiments and $P_{s}=0.79$ at 100 experiments (Figure 5C). From these simulated campaigns, we concluded that the FEA-informed approach should substantially accelerate optimization of toughness relative to the uninformative-prior approach.

It is worth noting that the experimental burden of the FEA methodology used herein is extremely low. The only input from experiment that is needed is a characterization of the materials. While we performed materials characterization by printing and evaluating cylindrical test coupons, for many relevant materials, high quality tabulated values exist. Further, the general method we developed to convert standard triangle language files appropriate for additive manufacturing to hexahedral meshes for FEA can be used for any general structure beyond the crossed barrel family. Thus, FEA for the simulation of resilience has a low barrier to entry and, with the continued growth of high performance computing resources, is likely to be an increasingly efficient path to gaining mechanical insight.

Based on the results of the simulated campaigns, six independent experimental campaigns were conducted to optimize toughness, each with an experimental budget of 60 experiments (Table 2). Three experimental campaigns used an uninformative prior and the remaining three used the FEA-informed approach. In contrast to what was observed for resilience (Figure 3D), the progression of the experimental response during each campaign was not a clear indicator of progress (Figure 5D), further emphasizing that a campaign's performance must be evaluated by assessing the predicted optimum design of the campaign. To directly evaluate these predicted optima, we carried out five repeated experiments on each predicted optimum after 20 and 60 experiments (Figure 5E). Here, the median and the range are plotted due to the large differences in toughness that arose for designs near the boundary of the imposed force threshold. While the acceleration observed for a particular campaign does in part depend on the location in parameter space of the first randomly selected experiment and the set of experimental responses observed for that particular campaign, the reduction in experiments observed in the experimental campaigns is comparable to reduction in experiments suggested by the simulated campaigns. In both simulated and experimental campaigns, the FEA-informed approach is superior to the uninformative-prior approach suggesting that, more generally, an optimization campaign benefits from the incorporation of FEA in the learning structure.

**Table 2:** Median and range of toughness after 60 experiments for both the uninformative-prior approach (not FEA-informed) and the FEA-informed approach

Approach	Median *U* (J)	Range (J)
Not FEA-informed	40.53	7.66
	35.91	16.25
	33.64	10.81
FEA-informed	50.70	13.18
	45.78	6.37
	32.92	20.60

To compare the performance of the approaches and accounting for the large range in performance for a given design, we computed the probability that a component designed by the FEA-informed approach would be tougher than a component designed by the uninformative prior approach (Figures S6A and S6B). Based on this metric, after 20 experiments, campaigns based on the FEA-informed approach had a 54% chance of producing tougher components that the uninformative-prior approach, which shows the two approaches are effectively equal at this stage. However, after 60 experiments, campaigns based on the FEA-informed approach had a 73% chance of producing tougher components than those produced by campaigns using an uninformative prior. Notably, after 20 experiments, the only campaign that identified a tougher component than the 35.4±1.5 J mean experimental response of the optimum found using the 600 measurements in a grid (Gongora et al., 2020) was a campaign based on the FEA-informed approach. Interestingly, after 60 experiments, four experimental campaigns, two with uninformative-prior approach and two with the FEA-informed approach, outperformed Grid, with the top performer being the FEA-informed approach outperforming Grid by ∼15%. Notably, the top performer discovered by this FEA-informed campaign was tougher than any we had previously identified in any experimental campaign. Ultimately, the FEA-informed approach outperformed the uninformative-prior approach by increasing the probability of finding a high-performing design after 60 experiments and reduced the number of experiments necessary to find a high-performing structure by 30-fold relative to Grid while increasing the performance by ∼15%.

### Conclusion

In this work, we used a case study in mechanics to explore several ways in which simulation data can be inserted into AE and evaluated the degree to which each accelerates research. Two mechanical properties were used: one, resilience, that can be robustly simulated; and one, toughness, that cannot. We found that when a good simulator exists for the property of interest, AE campaigns can be significantly accelerated using simulation knowledge as a Bayesian prior. This was demonstrated for the case of resilience, where a ∼10-fold reduction in the number of experiments was observed when FEA was incorporated in the belief model via discrepancy modeling versus a traditional BO approach. For toughness, in contrast, we developed a custom method for incorporating simulation in a BEAR by using FEA data to transform the space where belief modeling occurs and by using simulation to guide the decision policy. This custom method resulted in a ∼73% chance of outperforming a traditional BO approach with a ∼15% increase in component toughness. While the custom method developed in this work utilized ${\tilde{F}}_{y}$ in the AE system, FEA generally presents a valuable addition to the active learning component of an AE system, which is often viewed as purely data-driven. By capitalizing on the ability of FEA to predict certain properties of a system such as stresses and strains under varying loading or boundary conditions, myriad mechanical insights can be extracted and incorporated in the decision policy or the belief model to further accelerate the research process. Collectively, the principles described herein show how knowledge transfer from a simulator to an AE system may increase the pace of research not only in mechanics but also in other domains such as the physical sciences where simulation is ubiquitous but imperfect.

### Limitations of the study

The incorporation of simulation into AE in this work was explored using a BEAR in the context of mechanics where FEA was used to predict mechanical properties. To further explore the principles of knowledge transfer from simulation to AE, future studies need to focus on exploring the applicability and utility of these methods in other domains such as chemistry, biology, and materials science where simulation can be accessible.

### Resource availability

#### Lead contact

Further information and requests should be directed to and will be fulfilled by the lead contact, Keith A. Brown (brownka@bu.edu).

#### Materials availability

This study did not generate new unique reagents.

#### Data and code availability

All data needed to evaluate the conclusions in the paper are present in the paper and/or the supplemental information. The raw data can be accessed through www.kablab.org/data. Additional data related to this paper may be requested from the authors.

## Methods

All methods can be found in the accompanying transparent methods supplemental file.

## Supplemental information

Document S1. Transparent methods, Figures S1–S6, and Table S1
