# Supplementary material for: Using simulation to accelerate autonomous experimentation: A case study using mechanics
Source: iScience. 2021 Mar 2;24(4):102262. doi: 10.1016/j.isci.2021.102262 (PMC8010472; doi:10.1016/j.isci.2021.102262)
Supplement: Document S1. Transparent methods, Figures S1–S6, and Table S1 [file mmc1.pdf]

iScience, Volume *24*

Supplemental information

Using simulation to accelerate autonomous experimentation:

A case study using mechanics

Aldair E. Gongora, Kelsey L. Snapp, Emily Whiting, Patrick Riley, Kristofer G. Reyes, Elise F. Morgan, and Keith A. Brown

Supplemental Information

Transparent Methods:

Fabrication and Experimental Testing of Crossed Barrel Structures:

A crossed barrel structure with design parameters $x = (n, \theta, r, t)$, where n is the number of struts, θ is the angular displacement of the strut, r is the outer strut radius, and t is the thickness of the strut, was converted to a standard triangle language (STL) file using OpenSCAD. Using Slic3r, the generated STL file was converted to g-code and uploaded for fabrication to the MakerGear M3 FDM printers using OctoPrint. The crossed barrel structures were printed using polylactic acid (PLA) filament. The diameter of the printer nozzle was 0.35 mm and the diameter of the PLA filament was 1.75 mm. The nozzle temperature was 215 ℃, and the printer bed temperature was set to 75 ℃ for all layers after the first, which was printed with a 85℃ bed temperature. The structures were retrieved when the bed temperature was below 40 ℃, and then tested using uniaxial quasi-static compression on a universal testing machine (5965, Instron Inc.) at a speed of 3 mm/min with a force threshold of 4.8 kN. Toughness U was measured as the area under the force F-displacement D curve. The F-D curve was truncated if, after an initial displacement of 2 mm, the force was below 50 N. The height of the crossed barrel was 23 mm, and the outer and inner radius of the top disk was 13 and 8 mm, respectively. The fabrication process and testing protocol described up to this point was based upon our prior work (Gongora et al., 2020). Resilience U_E of the crossed barrel structure was measured as the area under the F-D up to the yield force, which was calculated from the 0.2% offset yield stress. When the yield force of the crossed barrel structure was larger than the force threshold, U_E was measured as the area under the entire F-D curve.

Finite-Element Analysis (FEA) of Crossed Barrel Structures:

To predict the resilience $\widetilde{U}_E$ and the yield force $\widetilde{F}_y$ of the crossed barrel structures, simulations of uniaxial quasi-static compression were performed with the finite element analysis (FEA) software package ABAQUS/Standard on the full-size model. The generated STL structure of a crossed barrel structure with design parameters $x = (n, \theta, r, t)$ was converted to a binary voxelized volume representation with voxels of length 0.25 mm where each voxel was then converted to an 8-node hexahedral finite element (C3D8 brick element) (**Figure S1**). The element size was selected to balance computational time and accuracy. To capture the response of PLA in FEA, an isotropic elastic, perfectly-plastic material model was used with Young's modulus $E =$ 1.66 GPa, yield strength $\sigma_y = 56.62$ MPa, and Poisson's ratio ν = 0.36. The E and σ_y of PLA used in the simulation were determined from stress σ-strain ε curves from uniaxial quasi-static compression tests of cylindrical samples of 8 mm diameter and 16 mm height, where E was measured from the slope of the σ-ε curve and σ_y was measured as the stress at the 0.2% offset strain (**Figure S2 A**). Based on 12 compression tests on identically prepared samples (**Figure S2 B**), the mean measurements of E and σ_y were used in FEA where the coefficient of variation (CV) of E and σ_y was 8.9% and 10.5%, respectively (**Figure S2 C**). To increase the throughput of

simulations, the simulations were conducted using a custom MATLAB script that automated the aforementioned meshing and analysis and ran on Boston University's shared computing cluster (SCC). To capture FEA predictions throughout parameter space, we built a surrogate model using Gaussian process regression trained using FEA calculations selected on a grid (**Figure S3**). The surrogate model for resilience had a root mean squared error (RMSE) of 0.0242 J and mean squared percentage error (MSPE) of 0.07% (**Figure S4 A**). The trained surrogate model for yield force had a RMSE of 0.0382 kN and MSPE of 0.02% (**Figure S4 B**).

Bayesian Optimization Formulation:

In the Bayesian optimization (BO) framework, we used a Gaussian process (GP) to build a belief model $\mathcal{B}_i = GP\left(\mu_i(x), \sigma_i^2(x)\right)$ with mean $\mu_i(x)$ and variance $\sigma_i^2(x)$ from i experimental observations $\{x_{1:i}, y_{1:i}\}$ to model the property of interest in the design space $x = (n, \theta, r, t)$. In particular,

$$\sigma_i^2(x) = \Sigma(x, x) - \Sigma(x, x_{1:i})(\Sigma(x_{1:i}, x_{1:i}) + \lambda^2 I_i)^{-1}\Sigma(x_{1:i}, x), \quad (1)$$

where λ defines the homoscedastic noise and I_i is the identity matrix. The covariance kernel $\Sigma(x, x')$ was a squared exponential, specifically,

$$\Sigma(x, x') = \alpha^2 \exp\left(-\frac{1}{2}\sum_{j=1}^{d}\left(\frac{\left(x_j - x'_j\right)^2}{\beta_j^2}\right)\right). \quad (2)$$

The kernel was parametrized by $d + 1$ parameters (specifically α and β_j, the latter of which comprises d values) where the design space dimensionality $d = 4$. The parameters of the kernel and the noise were optimized using maximum likelihood estimation after every subsequent experiment. Additionally, the hyperparameters were bounded to be greater than or less than their initialization values by at most a factor of 100 to avoid extremal hyperparameters or over-fitting.

Learning was performed using experimental resilience U_E, experimental toughness U, FEA-calculated resilience $\widetilde{U}_E(x)$, and FEA-calculated yield force $\tilde{F}_y(x)$. In the case of using an FEA-prior approach to optimize U_E, we defined a discrepancy model $\delta(x) = U_E(x) - \widetilde{U}_E(x)$ to explicitly learn the difference between experiment and FEA. In the case of using an FEA-informed approach to optimize U, we defined an effective length $L(x) = \frac{U(x)}{\tilde{F}_y(x)}$ to conceptually separate effects from strength and ductility. In campaigns that were not informed by FEA, a zero-mean prior was used. For optimizing toughness, we defined a logistic function P_F to filter parameter space. Specifically, $\mathrm{P}_F = 1$ indicated that $\tilde{F}_y(x)$ would not exceed the force threshold $F_t = 4.8$ kN and $\mathrm{P}_F = 0$ indicated that $\tilde{F}_y(x)$ would exceed F_t. Additionally, a transition region with a width of 15%, a number based on the coefficient of variation (CV) of U, was defined to account for structures with $\tilde{F}_y(x)$ near F_t. The logistic regression function was built from FEA and the force threshold as,

$$\mathrm{P}_F(x) = \frac{1}{1 + \exp\left(k\left(\tilde{F}_y(x) - F_t\right)\right)}, \quad (3)$$

with $k = 6.396$ to produce a 15% width.

Experimental campaigns began with an experiment that was selected uniformly at random in parameter space. Subsequent experiments were selected based upon an expected improvement (EI) decision policy. Specifically, after i experiments, the mean and variance of the belief model were calculated based on Table S1 and Equation (1), respectively. The decision policy used for each campaign is given in Table S1.

Table S1. Mean and decision policy used in each of the experimental and simulated campaigns, related to Figures 3 and 5.

Campaign	Mean $\boldsymbol{\mu_i(x)}$	Decision policy $\boldsymbol{x_{i+1}}$
FEA-informed resilience	$\widetilde{U}_E(x) + \Sigma(x, x_{1:i})(\Sigma(x_{1:i}, x_{1:i}) + \lambda^2 I_i)^{-1}\left(U_E(x_{1:i}) - \widetilde{U}_E(x_{1:i})\right)$	$argmax(EI(x)\vert\mathcal{B}_i)$
Resilience without FEA	$\Sigma(x, x_{1:i})(\Sigma(x_{1:i}, x_{1:i}) + \lambda^2 I_i)^{-1}(U_E(x_{1:i}))$	$argmax(EI(x)\vert\mathcal{B}_i)$
FEA-informed toughness	$\widetilde{F}_y(x) * \Sigma(x, x_{1:i})(\Sigma(x_{1:i}, x_{1:i}) + \lambda^2 I_i)^{-1}\left(\frac{U(x_{1:i})}{\widetilde{F}_y(x_{1:i})}\right)$	$argmax(EI(x) \cdot P_F(x)\ \vert\mathcal{B}_i)$
Toughness without FEA	$\Sigma(x, x_{1:i})(\Sigma(x_{1:i}, x_{1:i}) + \lambda^2 I_i)^{-1}(U(x_{1:i}))$	$argmax(EI(x)\vert\mathcal{B}_i)$

Simulated learning campaigns were conducted to assess the performance P of the various approaches to optimize U_E or U. The experimental observations used in the simulations were drawn from a dataset y_{600} comprised of the mean U_E or U for 600 distinct designs where each of the 600 designs was fabricated and tested in triplicate for a total of 1,800 experiments. To test a given combination of learning approach and campaign goal, a total of 100 independent simulated learning campaigns were conducted where the initial experiment for each simulated campaign was selected uniformly at random from the dataset. In the simulated learning campaigns, the subsequent experiments were selected from y_{600} using EI as described in Table S1. The performance of a given campaign after i experiments was given by its predicted optimum x_i^* and was defined as $P_i = \frac{y_{600}(x_i^*)}{\max(y_{600})}$. Zero-mean Gaussian noise was also added to the experimental observations with SD equal to the median SD of the dataset, 0.09 J for U_E and 1.66 J for U. Based on the P of 100 independent simulations, the probability of success as a function of i was calculated as $P_s = \frac{\sum_{j=1}^{100} \phi(P_{i,j})}{100}$ where $\phi(P_{i,j}) = 1$ if $P_{i,j} \geq 0.90$ and $\phi(P_{i,j}) = 0$ otherwise. These simulations were conducted in MATLAB using the Statistics and Machine Learning Toolbox.

To evaluate the degree to which the FEA simulation would deviate if the material properties were set to incorrect values, we predicted resilience $\widetilde{U}_E$ with varying E and σ_y. Specifically, we increased and decreased E by 5%, 10%, and 50%, while keeping σ_y constant. Additionally, we repeated this process for σ_y while keeping E constant. From this study, we found that both properties linearly affected $\widetilde{U}_E$ with a 1% increase in E resulting in a 0.36% decrease in $\widetilde{U}_E$ and a 1% increase in σ_y resulting in a 1.36% increase in $\widetilde{U}_E$. To investigate the performance of

the FEA-informed approach in the presence of variable input parameters that affect the FEA predictions, we conducted simulating learning campaigns in which FEA predictions were multiplied by a factor ϕ_{FE} = 0.50, 0.90, 0.95, 1.05, 1.1, and 1.5 (**Figure S5**). The range of ϕ_{FE} was selected to reflect the variability introduced when incorrect material properties are employed that would skew the FEA predictions underestimating or overestimating the experimental resilience U_E. From these simulations, we observed that within 10% (ϕ_{FE} = 0.90, 0.95, 1.05, and 1.1), the performance P of the FEA-informed approach varied slightly from the FEA-informed approach used in the study (ϕ_{FE} = 1). For ϕ_{FE} = 0.5, a small deviation was observed in P while for ϕ_{FE} = 0.90 a large deviation was observed in P. In totality, these results support that while variable material properties can affect FEA predictions, the FEA-informed approach still outperforms the uninformed-prior approach even when very inaccurate material properties are used.

Six independent experimental campaigns were conducted using the BEAR for U_E or U with three campaigns using the uninformative-prior approach and three campaigns using an FEA-based approach, namely the FEA-prior for U_E and the FEA-informed approach for U. The first experiment was selected uniformly at random, and the campaigns ran for the corresponding allotted experiment budget without a human in the loop. In the formulation of the decision policy, subsequent experiments were selected from a uniformly random, finite number of candidate designs drawn from parameter space. In the uninformed-prior approach for optimizing U_E and U, the predicted optimum after i experiments was selected as $x_i^* = \text{argmax}(\mu_i(x))$. In the FEA-prior approach to optimize U_E, the optimum structure also selected as $x_i^* = \text{argmax}(\mu_i(x))$. In the FEA-informed approach to optimize U, the predicted optimum structure at i experiments was selected as $x_i^* = argmax\left(\mu_i\left(x_{P_F \geq 0.90}\right)\right)$, where $x_{P_F \geq 0.90}$ was the subset of designs in parameter space $P_F \geq 0.90$. This was done to account for areas in parameter space near the force threshold, where there is a sharp decrease in U. The probability of an FEA-informed structure outperforming the uninformative approach was computed by calculating the difference between the FEA evaluation measurement and the uninformative-prior measurement and assigning 1 if the difference was positive and 0 otherwise. The probability was then computed for each structure, and the average probability was calculated (**Figure S6 A and B**).

Supplemental Information:

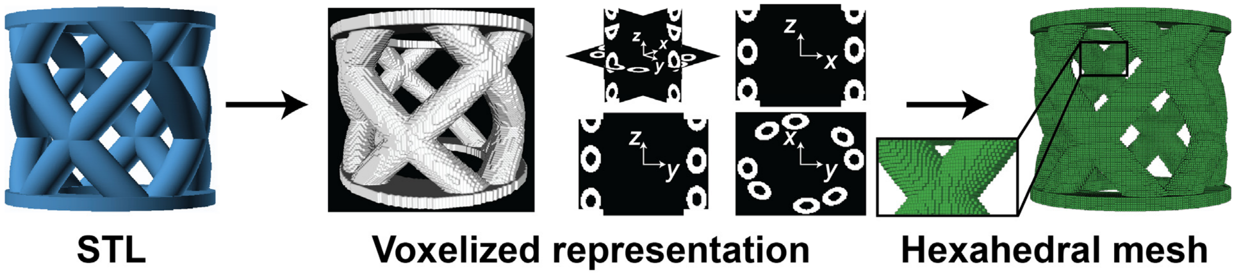

Figure S1: The standard triangle language (STL) file of each crossed barrel structure was first converted to a voxelized representation and then the individual voxels were converted to 8-node hexahedral finite-elements, related to Figure 2.

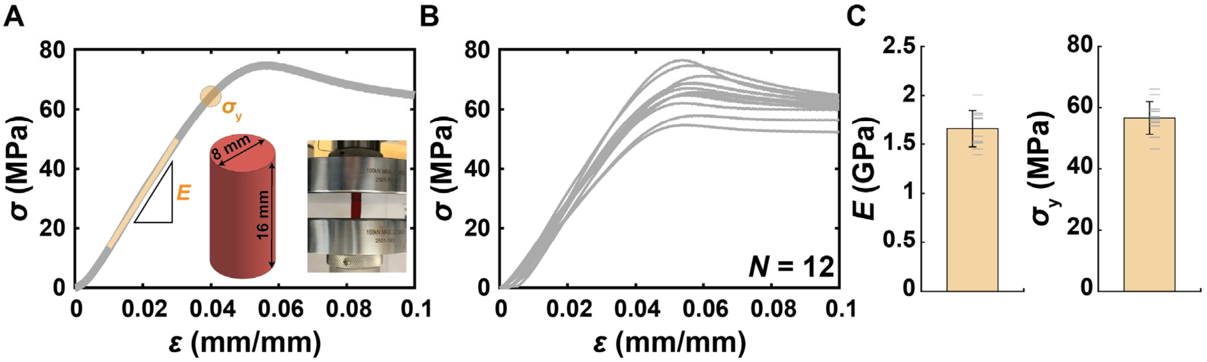

Figure S2: Material properties of polylactic acid (PLA), related to Figure 2.

A. **Stress σ-strain ε curve showing uniaxial compression testing of a cylindrical specimen that was 8 mm in diameter and 16 mm tall. This curve was used to determine Young's modulus E and yield strength σ_y.**
B. **Measurements of E and σ_y obtained from the compression tests of 12 cylindrical specimens.**
C. **From the measurements of E and σ_y , the mean (height of the bar) and standard deviation (error bars) were determined. The individual measurements are shown as grey ticks.**

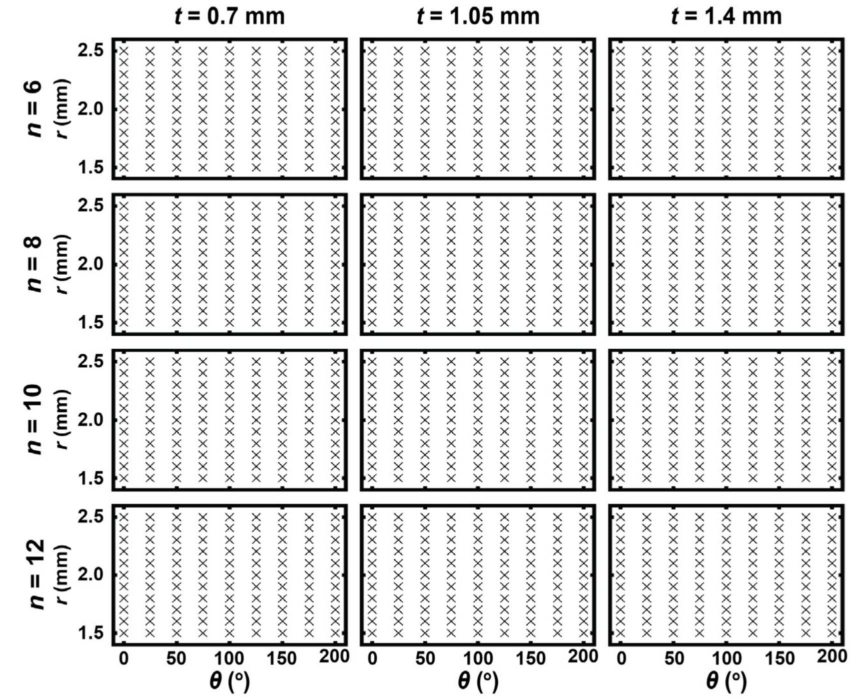

Figure S3: Markers indicate the 1,188 design locations sampled by grid-search to obtain finite-element analysis (FEA) predictions from which to build a surrogate model using gaussian process regression, related to Figures 3 and 5.

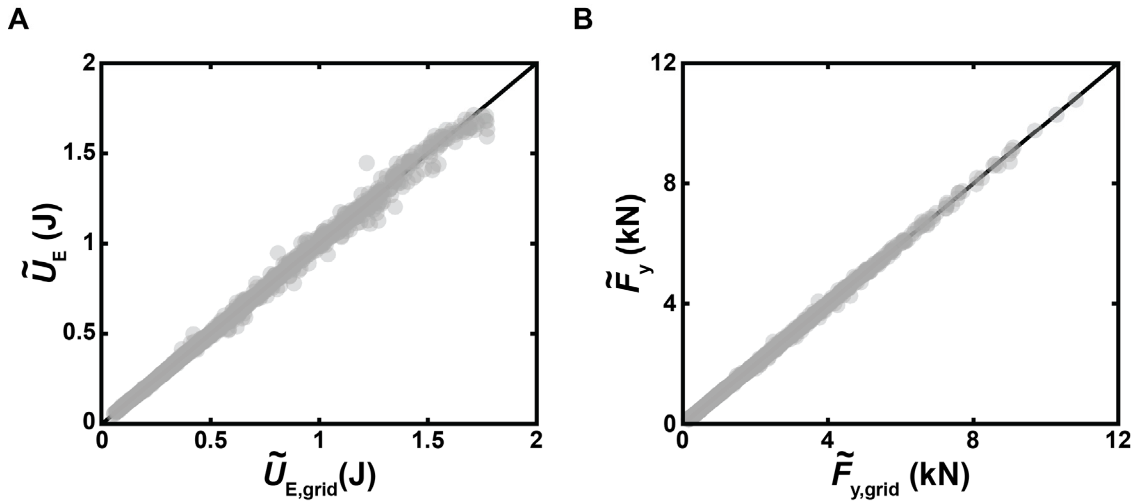

Figure S4: Parity plots of surrogate model predictions versus FEA observations from grid-search, related to Figures 3 and 5.

A. Surrogate model predictions of resilience $\widetilde{U}_E$ vs. FEA observations from grid-search $\widetilde{U}_{\mathrm{E,grid}}$.

B. Surrogate model predictions of yield force $\widetilde{F}_y$ vs. FEA observations from grid-search $F_{\mathrm{y,grid}}$.

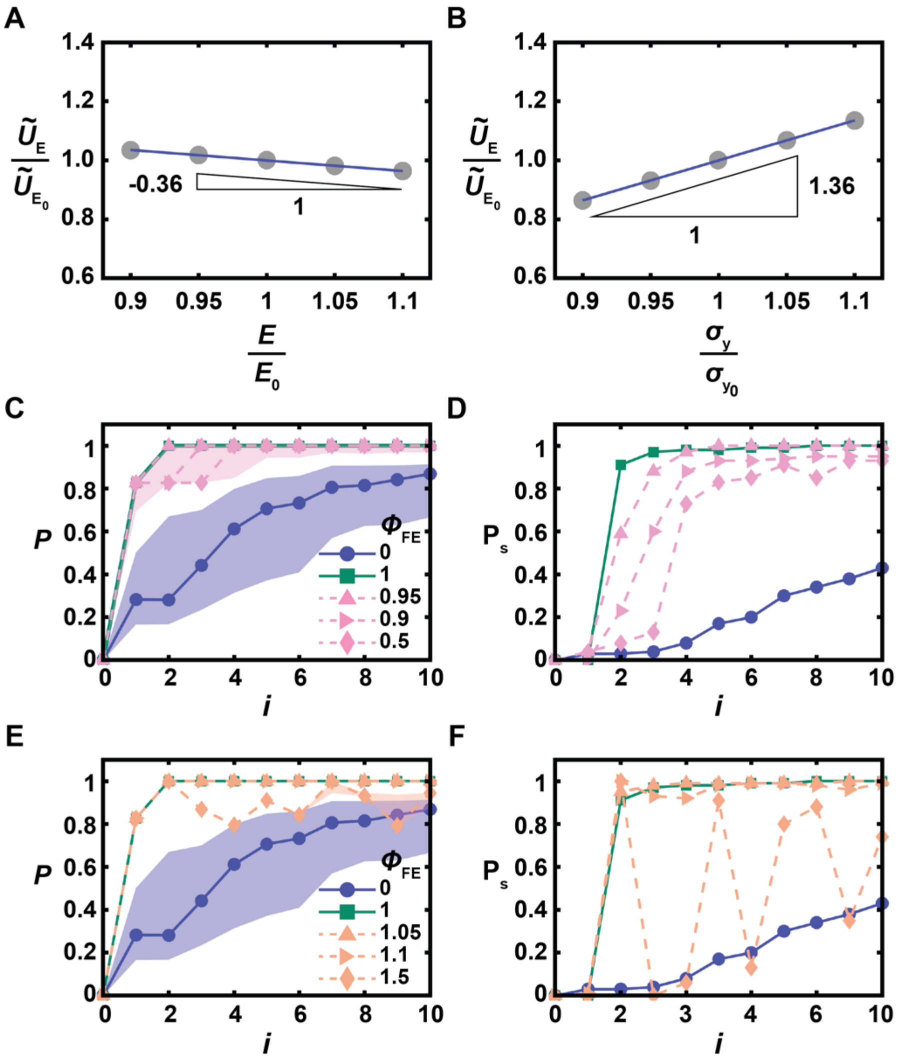

Figure S5: Performance of simulated learning campaigns for resilience U_E with varying finite element analysis (FEA) prediction quality, related to Figure 3.

A. **FEA predicted resilience $\tilde{U}_E$ vs. Young's modulus E where $\tilde{U}_{E_0}$ is the FEA prediction using the experimentally determined Young's modulus E_0 and yield strength σ_{y_0}.**

B. **$\tilde{U}_E$ vs. yield strength σ_y.**

C. **Simulated performance P at experiment number i for a simulated learning campaign to optimize resilience using FEA predictions varied with multiplication factor ϕ_{FE} where $\phi_{FE} = 0$ corresponds to the uninformative prior approach, $\phi_{FE} = 1$ corresponds to the FEA-informed approach used in the study, and ϕ_{FE} = 0.95, 0.9, and 0.5 correspond to decreasing the FEA predictions.**

D. **Probability P_s of achieving $P \geq 0.90$ at a given i for ϕ_{FE} = 0, 0.5, 0.9, 0.95, and 1.**

E. **P at i for a simulated learning campaign to optimize resilience using FEA predictions varied with ϕ_{FE} where ϕ_{FE} = 1.05, 1.1, and 1.5 correspond to increasing the FEA predictions.**

F. **P_s of achieving $P \geq 0.90$ at a given i for ϕ_{FE} = 0, 1, 1.05, 1.1, and 1.5.**

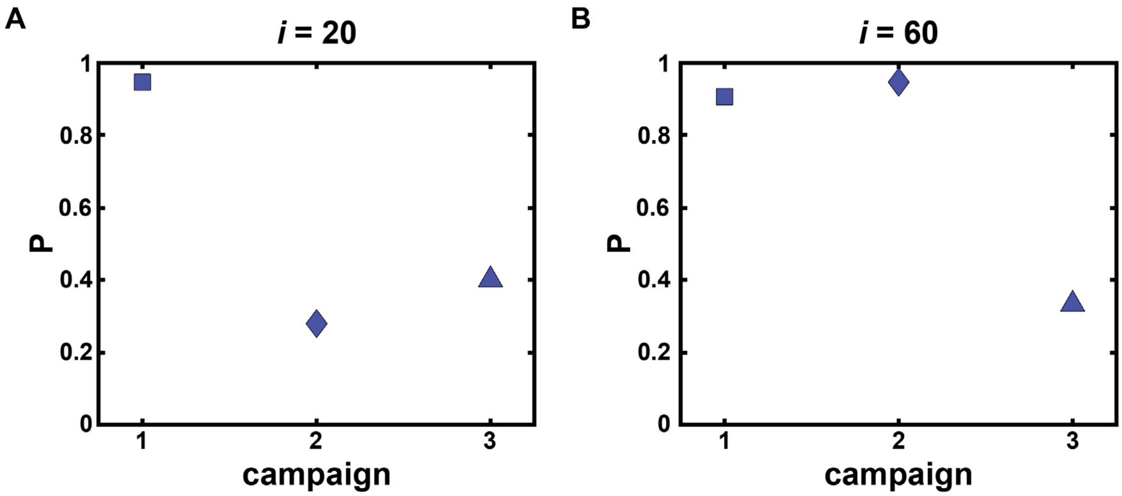

Figure S6: Experimentally determined probability that an FEA-informed campaign will identify a design with higher toughness U than that identified by an uninformative-prior campaign after $i\ = 20$ experiments (A) and $i = 60$ experiments (B), related to Figure 5.
